# OP13 Improving Case Finding For Celiac Disease In Children And Adults: Evidence Synthesis And Economic Modeling

**DOI:** 10.1017/S0266462324000771

**Published:** 2025-01-07

**Authors:** Martha Elwenspoek, Howard Thom, Athena Sheppard, Edna Keeney, Rachel O’Donnell, Joni Jackson, Cristina Roadevin, Sarah Dawson, Deborah Lane, Jo Stubbs, Hazel Everitt, Jessica Watson, Alastair Hay Peter Gillett, Gerry Robins, Hayley Jones, Sue Mallett, Penny Whiting

## Abstract

**Introduction:**

Celiac disease (CD), an autoimmune disorder triggered by gluten, impacts about one percent of the population. Only one-third receive a diagnosis, leaving the majority unaware of their condition. Untreated CD can lead to gut lining damage, resulting in malnutrition, anemia, and osteoporosis. Our primary goal was to identify at-risk groups and assess the cost-effectiveness of active case finding in primary care.

**Methods:**

Our methodology involved systematic reviews and meta-analyses focusing on the accuracy of CD risk factors (chronic conditions and symptoms) and diagnostic tests (serological and genetic). Prediction models, based on identified risk factors, were developed for identifying individuals who would benefit from CD testing in routine primary care. Additionally, an online survey gauged individuals’ preferences regarding diagnostic certainty before initiating a gluten-free diet. This information informed the development of economic models evaluating the cost-effectiveness of various active case finding strategies.

**Results:**

Individuals with dermatitis herpetiformis, a family history of CD, migraine, anemia, type 1 diabetes, osteoporosis, or chronic liver disease showed one and a half to two times higher risk of having CD. IgA tTG, and EMA demonstrated good diagnostic accuracy. Genetic tests showed high sensitivity but low specificity. Survey results indicated substantial variation in preference for certainty from a blood test before initiating a gluten-free diet. Cost-effectiveness analyses showed that, in adults, IgA tTG at a one percent pre-test probability (equivalent to population screening) was the most cost effective. For non-population screening strategies, IgA EMA plus HLA was most cost effective. There was substantial uncertainty in economic model results.

**Conclusions:**

While population-based screening with IgA tTG appears the most cost effective in adults, decisions for implementation should not solely rely on economic analyses. Future research should explore whether population-based CD screening aligns with UK National Screening Committee criteria and requires a long-term randomized controlled trial of screening strategies.

